# Guideline for the management of pediatric off-label use of drugs in China (2021)

**DOI:** 10.1186/s12887-022-03457-1

**Published:** 2022-07-23

**Authors:** Min Meng, Enmei Liu, Bo Zhang, Quan Lu, Xiaobo Zhang, Bin Ge, Ye Wu, Li Wang, Mo Wang, Zhengxiu Luo, Ziyu Hua, Xiaoling Wang, Wei Zhao, Yi Zheng, Xinan Wu, Ruiling Zhao, Wenbo Meng, Li Xiang, Gang Wang, Yuntao Jia, Yongchuan Chen, Xiaoyan Dong, Lina Hao, Chengjun Liu, Meng Lv, Xufei Luo, Yunlan Liu, Quan Shen, Wenjuan Lei, Ping Wang, Yajia Sun, Juanjuan Zhang, Ling Wang, Ruobing Lei, Tianchun Hou, Bo Yang, Qiu Li, Yaolong Chen

**Affiliations:** 1 Department of Pediatric Research Institute, Children’s Hospital of Chongqing Medical University, Chongqing, 400014 China; 2 National Clinical Research Center for Child Health and Disorders, Ministry of Education Key Laboratory of Child Development and Disorders, China International Science and Technology Cooperation Base of Child Development and Critical Disorders, Children’s Hospital of Chongqing Medical University, Chongqing, 400014 China; 3 Chongqing Key Laboratory of Pediatrics, Chongqing, 400014 China; 4 Gansu Provincial Hospital, Lanzhou, 730000 China; 5 Department of Pharmacy, Peking Union Medical College Hospital, Beijing, 100730 China; 6 Department of Pulmonology, Shanghai Children’s Hospital, School of Medicine, Shanghai Jiao Tong University, Shanghai, 200040 China; 7 Children’s Hospital of Fudan University, Shanghai, 201102 China; 8 Peking University First Hospital, Beijing, 100034 China; 9 Peking University, Beijing, 100871 China; 10 Beijing Children’s Hospital, Capital Medical University, Beijing, 100045 China; 11 Shandong University, Jinan, 250100 China; 12 The First Hospital of Lanzhou University, Lanzhou, 730000 China; 13 Children’s Hospital of Shanxi, Taiyuan, 030012 China; 14 The First Affiliated Hospital of Third Military Medical University (Army Medical University), Chongqing, 400038 China; 15 Children’s Hospital Affiliated to Shandong University, Jinan, 250022 China; 16 School of Public Health, Lanzhou University, Lanzhou, 730000 China; 17 Evidence-Based Medicine Center, School of Basic Medical Sciences, Lanzhou University, Lanzhou, 730000 China; 18 Research Unit of Evidence-Based Evaluation and Guidelines, Chinese Academy of Medical Sciences (2021RU017), School of Basic Medical Sciences, Lanzhou University, Lanzhou University Institute of Health Data Science, Lanzhou, 730000 China; 19 Lanzhou University Institute of Health Data Science, Lanzhou, 730000 China; 20 WHO Collaborating Centre for Guideline Implementation and Knowledge Translation, Lanzhou, 730000 China; 21 Lanzhou University GRADE Center, Lanzhou, 730000 China

**Keywords:** Off-label use of drugs, Management, Children, Guideline

## Abstract

**Background:**

The "Law on Doctors of the People's Republic of China," which was officially implemented on March 1, 2022, emphasizes the requirements for rational drug use and the necessity for appropriate management of off-label drug use. The safety and ethical considerations related to off-label drug use are different in children than in adults. There is so far no management guideline for pediatric off-label use of drugs in China, and the applicability of foreign guidelines is limited. Establishing a localized evidence-based management guideline for pediatric off-label use of drugs to support the national legislation and clinical practice is of critical importance.

**Methods:**

We established a guideline working group, including experts from a broad range of disciplines and developed recommendations following the guidance of the World Health Organization Handbook and the Chinese Medical Association. The following themes were identified by questionnaires and expert interviews to be of great concern in the management of off-label drug use in children: general principles and characteristics of management of pediatric off-label drug use; establishment of expert committees; evidence evaluation; risk–benefit assessment; informed consent; monitoring and assessment of the risk; and monitoring and patient education. Two rounds of Delphi surveys were organized to determine the final recommendations of this guideline. We graded the recommendations based on the body of evidence, referring to the evaluation tool of the Evidence-based management (EBMgt) and the Oxford Center for Evidence-Based Medicine: Level of Evidence (March 2009).

**Results:**

We developed the first guideline for the management of pediatric off-label use of drugs in China.

**Conclusions:**

The guideline is to offer guidance for pediatricians, pharmacists, medical managers, policymakers, and primary care physicians on how to manage off-label drug use in pediatrics and to provide recommendations for Chinese healthcare policy in the future.

**Supplementary Information:**

The online version contains supplementary material available at 10.1186/s12887-022-03457-1.

## Background

Off-label use refers to the use of drugs beyond the scope of the drug instructions and labels provided by the manufacturer and approved by the National Medical Products Administration of China [1]. Different forms of off-label use include the use of drugs for unapproved indications, unapproved dosage or frequency, unapproved route of administration, or the use for unapproved population (e.g. age group). Off-label use of drugs is common in clinical practice [2–4], especially in children, with the proportion of children who were prescribed drugs off-label ranging from 3.2% to 95% [2, 3]. However, there is a lack of evidence to support pediatric off-label use of drugs [4]. In 2015, an expert consensus statement on the off-label use of drugs was published by the Drug Risk Management Group of the Professional Committee for Therapeutic Drug Monitoring and Research, Chinese Pharmacology Association [5]. Subsequently, in 2016 the Chinese Society of Pediatric Clinical Pharmacology and the Chinese Medical Association published an expert consensus statement on off-label drug use in children in China [1]. In the last five years, along with the new evidence that has become available, standards, norms, and consensus statement related to pediatric off-label use of drugs have been updated. In 2020, a joint policy statement by the European Academy of Pediatrics and the European Society for Developmental Perinatal and Pediatric Pharmacology on the off-label use of drugs in neonates, infants, children and adolescents [4] was published, which, however, did not include evidence from China as they intentionally restricted their body of evidence to Europe. Chinese consensus statements or guidelines on off-label drug use, such as the Catalogue of Off-label Usage (The New Usage of 2020) developed by the Guangdong Pharmaceutical Association [6], have not provided recommendations for off-label use of drugs in children. In response to these issues, the Chinese Society of Pediatric Clinical Pharmacology, the Chinese Medical Association, and the National Clinical Research Center for Child Health and Disorders (Children's Hospital of Chongqing Medical University) in collaboration with the Chinese GRADE Center, developed the present guideline, Guidelines for the Management of Pediatric Off-label Use of Drugs in China, which aims to promote the rationalization and standardization of off-label use of drugs in children, and ultimately ensure the safety of drugs in the treatment of children.

## Methods

This guideline was developed by the Chinese Society of Pediatric Clinical Pharmacology, the Chinese Medical Association, the National Clinical Medical Research Center for Child Health and Disease (Children's Hospital of Chongqing Medical University), and the Chinese GRADE Center. Methodological support was provided by the Chinese GRADE Center, the World Health Organization (WHO) Collaborating Center for Guideline Implementation and Knowledge Translation, and the Institute of Health Data Science, Lanzhou University. The guideline was launched on April 12, 2021, and finalized on December 24, 2021. The guideline followed the methodology proposed by WHO [7] and the Chinese Medical Association [8], as well as the Appraisal of Guidelines for Research and Evaluation II (AGREE II) [9] and Reporting Items for Practice Guidelines in Healthcare (RIGHT) statement [10–12].

### Establishment of the guideline working group

Experts from a broad range of disciplines including pediatrics, pharmacy, management and health policy, and evidence-based medicine, as well as parents of children participated in the development of the guidelines. The participants were divided into the steering committee, a secretarial team, an evidence team, a consensus team, and an external review team. A full list of members is provided at the end of this article. All members completed a declaration of interest form and stated that they had no economic conflicts of interest directly relevant to this guideline.

### Registration of the guideline

This guideline is registered at the International Practice Guideline Registry Platform (Registration no. IPGRP- 2021CN088). The guideline protocol is available on request from the registration platform.

### Target users and population of the guideline

The target audience of this guideline includes pediatricians, pharmacists, medical managers, policymakers, and primary care physicians. The management population of the guideline is children and their guardians.

### Determination of essential themes

The themes of this guideline were determined during one round of a questionnaire-based survey and two rounds of expert interviews. At the beginning, the working group identified 11 themes by analyzing published guidelines relevant to the topic and interviewing pharmaceutical specialists. Subsequently, we developed a questionnaire to evaluate the importance of these themes and ask suggestions for additional themes. Sixty-six themes were obtained through the feedback from the survey. After de-duplication, merging, and discussion with the consensus team meeting, eight themes were finally selected to be included.

### Evidence search and screening

We performed a systematic literature search for each question through PubMed, Web of Science, Embase, China Biomedical Literature Database (CBM), WanFang Data, and China Knowledge Network database (CNKI) to identify literature published from inception to August 13, 2021. We used the search terms "pediatric*", "paediatric*", "child*", "infant*", "adolescent*", "neonat*", "newborn*", "teen*", "off-label", "off label use*", "unlabeled use*" and "unlicensed medicines" (Supplementary file 1). Google Scholar and reference lists of the studies identified in the database search were also searched to avoid missing potentially relevant literature. We made no restrictions on study design. Only studies published in English and Chinese were included. After the literature search, the sixteen evidence team members were divided into eight pairs; each pair was assigned one theme. Each pair searched literature relevant for their target theme, after which each potentially relevant record was evaluated by both investigators independently, and finally information related to their target theme was extracted from the eligible studies. Disagreements in study selection were resolved by joint discussion or consultation with a third researcher.

### Grading the quality of evidence

The evidence team graded the quality of evidence and the strength of each recommendation using the Evidence-based Management evaluation tool (EBMgt) [12] combined with the Oxford Center for Evidence-Based Medicine (OCEBM, 2009) [13] (see Table 1). We also considered the relevant laws and regulations in China when making the appraisal.

**Table 1:** Classification of evidence quality and strength of recommendations

Strength of recommendation	Quality of evidence	Content
A	1	RCTs or meta analyses
B	2	High-quality, repeatable and comprehensive literature reviews, providing evidence synthesis and operability recommendations, or a systematic review
	3	Multi-center data comparative studies, multi-center case studies or large sample quantitative studies
C	4	Small sample, single center qualitative or quantitative studies
	5	Descriptive studies and/or case reports, usually including observations, warnings and recommendations for managers
D	6	Opinions of authoritative experts or expert committees, generally based on expert experience

### Formation of recommendations

Based on the summary table of the available evidence for each theme and consideration of patients' preferences and values, fairness, and the implementation ability of the management measures, we drafted initial recommendations on off-label drug use for children. Two rounds of Delphi surveys on recommendations were then conducted on November 4, 2021, and November 8, 2021, respectively, and a total of 19 suggestions from experts were collected. Recommendations were considered to have reached a consensus when 80% of the voters agreed to the recommendation.

### Writing and external review of the guideline

The secretariat prepared the first version of the guideline in accordance with the RIGHT checklist and sent the full text to the external review committee. All opinions and recommendations of the review committee were taken into consideration in the revision and creation of the final draft of the guideline.

### Updating the guideline

This guideline is planned to be updated within three to five years based on emerging relevant evidence, following the Reporting Items for Updated Clinical Guidelines: Checklist for the Reporting of Updated Guidelines (CheckUp [14]) checklist.

## Recommendations

### Theme 1: general principles and characteristics of pediatric off-label use of drugs management

#### Recommendation 1.1 It is recommended that pediatric off-label use of drugs should be managed in accordance with the general principles of the "Management Guideline for the Off-label Use of Drugs in China (2021)". (2; B)

The guideline entitled "Management Guideline for the Off-label Use of Drugs in China (2021)" focuses on the definition of off-label use of drugs, evidence grading standards, management of informed consent, monitoring of adverse drug reactions and adverse events, main responsibilities of hospitals and manufacturers, conditions for reimbursement by social health insurance, and the national approval system, resulting in recommendations in nine thematic areas (Supplementary file 1). Pediatric off-label drug use should adopt the general management principles in adults adapted according to the specific characteristics of the child population.

#### Recommendation 1.2 Clinicians are recommended to refer to the published guidelines or consensus statements on the specific topic for children.(2; B)

Article 29 of the Law on Doctors of the People's Republic of China in 2021 [16] states that "Physicians shall adhere to the principles of safe, effective, economic, and reasonable drug use, and follow the guidelines for clinical application of drugs, clinical treatment guidelines, and drug instructions for rational drug use". The evidence team extracted and analyzed evidence-based recommendations for pediatric off-label use of drugs based on medical evidence by searching domestic and international guidelines or consensus statements on children (2018–2021) and developed a list of common types pediatric off-label use of drugs, evidence levels, and recommendations (Supplementary file 2). The list will be made available online and updated regularly by the Pediatric Clinical Evidence and Guidelines database of Children's Hospital of Chongqing Medical University.

### Theme 2: the establishment of an expert committee on the pediatric off-label use of drugs

#### Recommendation 2.1 We suggest that tertiary level A hospitals with pediatrics departments and children's specialty hospitals establish an expert committee on pediatric off-label drug use, which should be under the guidance of the pharmaceutical management and pharmacotherapy committee and/or ethics committee. (6; D)

A multicenter study revealed that more than half (55.4%) of health care workers did not follow the “Expert Consensus on Off-label drugs in pediatrics in China” guidance document, with 52.6% of the reasons being "no expert group on off-label use" [12].

#### Recommendation 2.2 We suggest that two experts, a clinical expert and a clinical pharmacist, co-chair the expert committee. (3; B)

To ensure the rationality of off-label use of drugs to treat children, the process of protocol development and safety monitoring requires the discussion and collaboration of experts from various disciplines, including pediatricians, pharmacists, and nurses [15–17]. In addition, pediatric clinical specialists and clinical pharmacologists play an essential role in decision-making regarding pediatric off-label use of drugs [18, 19].

#### Recommendation 2.3 If conditions permit, we suggest expanding the diversity in the experience among the members of the expert group with the following fields: evidence-based medicine, health economics, epidemiology, ethics and law. (3; B)

Guidelines and consensus statements on the management of off-label use of drugs for children from China and abroad emphasize that off-label drug use should be evidence-based. Most clinicians, however, lack the necessary skills and time to conduct systematic reviews of existing evidence [20]. Evaluating the body evidence for off-label drug use is particularly complicated for certain groups of children, such as preterm infants, newborns, infants and children below the age of two years, and children with chronic or rare diseases [21–23]. Furthermore, the legal and ethical issues for off-label drug use are more complex in children than adults [24]. As a result, we suggest to expand the expert group's fields of expertise beyond the minimum level if possible.

#### Recommendation 2.4 If children have comorbidities or the simultaneous off-label use of multiple drugs is needed, we suggest to extend the expert group with

- **Clinical experts in the corresponding diseases.**
- **Non-clinical experts in medical technology, such as professionals in clinical testing, pathology, imaging, or ultrasound.**
- **Experts in the field of essential medicine, such as biologists and pharmacologists. (6; D)**

In complex clinical cases involving, the expert committee can be expanded if the expertise of the regular committee does not cover all aspects related to the actual situation of the patient. Under the principles of individualized medical model and precision medicine, we suggest that the expert groups should be expanded on a case-by-case basis to include non-clinical medical technologists and basic medicine specialists when discussing off-label treatment of children who have comorbidities or need to take multiple drugs off-label at the same time. For example, we suggest including microbiologists for questions regarding the off-label use of drugs to treat infectious diseases; and radiologists for questions related to ultrasound and MRI contrast agents in children.

#### Recommendations 2.5 The primary responsibilities and obligations of the expert group on the off-label use of drugs to treat children should include

- **Formulating and regularly updating the list of drugs commonly used off-label for children in their respective medical institution;**
- **Participate in consultations for off-label drug use in children;**
- **Promptly release data on serious adverse events or adverse reactions(ADRs) related to off-label drug use. (6; D)**

### Theme 3: evidence evaluation of off-label drug use for children

#### Recommendation 3.1 We suggest that the level of evidence and related recommendations for the use of off-label drugs should be formed following the "Guideline for the Off-label Use of Drugs in China". (2; B)

#### Recommendation 3.2 When direct evidence is unavailable, the three categories of indirect evidence listed below should be specifically investigated and evaluated. (2; B)

- **Evidence from adults can be used as indirect evidence;**

In the lack of high-quality pediatric clinical evidence, an extrapolation of evidence from adult trials can help mitigate the impact of limitations caused by insufficient sample size, disruption of equilibrium, and heterogeneity in pediatric clinical trials [25]. Both the Chinese Food and Drug Administration [26] and the US Food and Drug Administration (FDA) [27, 28] have proposed extrapolating adult evidence to pediatric populations by carefully assessing the indirectness of adult evidence, which is an effective method when high-quality evidence for pediatric populations is lacking. Therefore, this guideline draws on the requirements of the Chinese Technical Guidelines for the Extrapolation of Adult Data to Pediatric Populations (2017) [26] in China, which suggests assessing the indirectness of adult evidence.

We suggest assessing the evidence by systematic reviews in cases when there are not yet any approved indications for children, but there is evidence for both adults (in China) and children (anywhere).

When there exists evidence only from Chinese adults evidence and no high-quality evidence is available for the pediatric population, we suggest using these findings with caution. A comprehensive analysis of all available information and data is required prior to use, including differences in organ function in specific age groups, the impact on pharmacological characteristics, knowledge of the disease, epidemiological conditions, non-clinical trial data, pharmacokinetics (PK), pharmacodynamics (PD), clinical effectiveness, and safety differences between adult and pediatric populations for drugs with the same or similar mechanism.

- **Evidence from similar diseases in children can be used as indirect evidence;**

Evidence from similar diseases may need to be considered for some specific types of disease, such as rare diseases in children [29, 30] or public health emergencies [31]. For example, the development of the rapid guidelines for COVID-19 in children was informed by severe acute respiratory syndrome [31] (SARS), middle east respiratory syndrome coronavirus (MERS), and influenza studies as indirect evidence.

- **Evidence from pharmacokinetic or pharmacodynamic equivalency modeling studies on pediatric populations can be used as indirect evidence.**

It is not suitable to adopt the adult dose directly for children based on the child's age and weight alone [32]. Instead, after studying and assessing the facts on pharmacokinetics, inferences must be drawn. In the absence of high-quality evidence, researchers have used data from pharmacokinetic models of piperacillin and amikacin in critically unwell infants to recreate the concentration–time curve and provide evidence related to the optimal dosage for clinicians to employ in their everyday practices [33]. Alternatively, precise drug dosages for neonates and babies can be produced by integrating dose validation, population pharmacokinetics, and mathematical models of drug clearance and distribution, allowing for safer and more effective off-label use of drugs [34].

### Theme 4: benefit and risk assessment of off-label drug usese in children: steps and precautions

#### Recommendation 4.1 We suggest using the Benefit and Risk Assessment for Off-label Use (BRAvO) decision-making framework. (2; B)

**Fig. 1:**
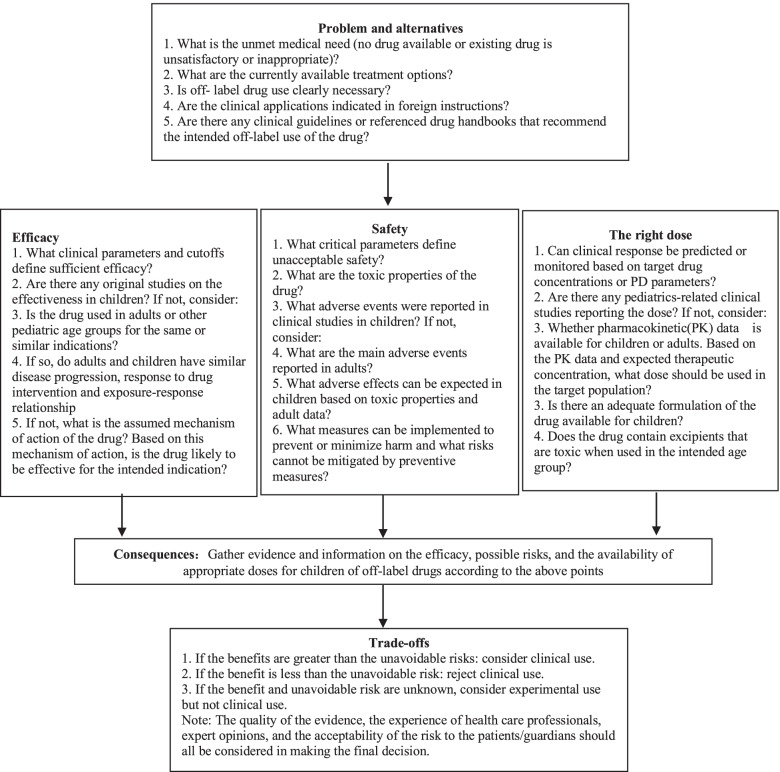
Framework for benefit-risk assessment of pediatric off-label use of drugs

Benefit and risk assessment is an appropriate method to judge the rationality of off-label drug use [4, 5]. In recent years, scholars in China and abroad have proposed many models and tools for benefit-risk assessment of drugs [35], such as the ProOACT-URL developed by the European Medicines Agency (EMA) [36] and the PhRMA Benefit-Risk Action Team (BRAT) framework [37]. In 2021, the Benefit and Risk Assessment for Off-label Use (BRAvO) decision framework was developed for children based on the EMA and BRAT frameworks [38]. The process in BRAvO is straightforward and it is therefore recommended as a decision framework for the benefit-risk assessment of off-label use of drugs in children (Fig. 1).

#### Recommendation 4.2.1 The risks associated with off-label use of drugs vary between different age groups of children. It is recommended that clinicians pay particular attention on how to treat neonates and preterm infants. (1; A)

The severe shortage of drugs for neonates is a worldwide problem [39, 40], mainly due to the extremely low percentage of clinical trials on medicines for neonates [41]. In children's general wards the proportion of children who were prescribed drugs off-label has been estimated between 18.2% and 70.0% [42]. Among neonates, the corresponding range was 26% to 95% [43], and in preterm infants, 95% to 100% [44, 45]. The younger the children, the fewer drug options are available [46]. Therefore, in the absence of strong evidence to support the best drug choice, neonatologists often rely on clinical experience, expert consensus, and evidence from adults and older children to infer the safest and most effective dosage and regimen, which has led to significant variation in the practice of neonatal drug use between and within countries [47].

#### Recommendation 4.2.2 Clinicians should consider the child’s age and the associated physiological, growth and development characteristics when prescribing drugs off-label. (5; C)

Compared with adults, assessing the benefit-risk of off-label use of drugs in children is more complicated [48]. Because of the immature excretory organs such as the liver and kidney, children have a low ability to metabolize and excrete drugs, resulting in a higher incidence of adverse drug reactions in children. The blood–brain barrier is more permeable in children, leading to easy deposition of drugs in brain tissue and causing neurological reactions [49]. In addition, children are in a stage of growth, with tissues, organs, and body composition, as well as the physiological characteristics, pharmacokinetics (PK), and pharmacodynamics (PD), constantly and rapidly developing [50]. These factors increase the difficulty of assessment of benefits and risks in the treatment of children. The adequate formulation and the safety of the excipients in the drug for children should be considered.

### Theme 5: informed consent for the off-label use of drugs in children. In general conditions (non-emergency disposal),

#### Recommendation 5.1 It is recommended that the guardians sign the informed consent for children under eight years of age. (2; B)

#### Recommendation 5.2 It is recommended that both the child and their guardians sign the informed consent for children over eight years of age and with sufficient autonomy. (2; B)

Informed consent is a crucial tool for doctors and patients to defend their legitimate rights and interests. Although children have a limited ability to estimate risks, signing informed permission is vital for children's mental health and treatment participation. Article 19 of the Civil Code of the People's Republic of China [51] stipulates that "A minor who has reached the age of eight is a person with limited capacity for civil conduct and shall be represented in the performance of civil juristic acts by his or her agent ad litem or obtain the consent or acknowledgment of his or her agent ad litem". Informed consent for clinical trials in children is recommended [52], with the age of 8 years serving as the criterion whether they should co-sign an informed consent with their guardians or not. Studies from abroad similarly recommend involving children with autonomous capabilities should participate in the informed consent process with their guardians if possible [53, 54]. Therefore, considering the situation and legislation in China, it is recommended that eight years of age be used as the age point for children to participate in informed consent. We suggest that the doctor in charge explain the reasons for off-label use adequately before signing an informed consent with the parent or child. The content of the informed consent was recommended as Sect.4.3 of the "Management Guideline for the Off-label Use of Drugs in China (2021)" (Supplementary file 1).

### Theme 6: evidence-based practice for the off-label use of drugs in children

#### Recommendation 6.1 We suggest to develop an evidence-based medicine database for off-label use drug use in children in China. (3; B)

Evidence-based drug databases and drug directories, such as the Micromedex Pharmaceutical Database, the British National Formulary for Children (BNF-c) [55], the Dutch National Formulary for Children [56], and the WHO Model Formulary for Children based on the Second Model List of Essential Medicines for Children [57], can effectively promote rational drug use in children [58, 59]. However, data from current international databases and catalogs are not necessarily applicable to Chinese children, especially for premature infants and newborns [4]. At present, there is no database on the off-label drug use for children in China, and the pediatric evidence-based drug databases or catalogs published by national and international societies tend to cover only a small number of drugs [60], have incomplete information, and be untimely updated. There is thus an urgent need for pediatric clinicians to establish a pediatric children's off-label drug database [12, 61]. To promote the standardized and science-based off-label use of drugs in children, we suggest establishing a user-friendly, evidence-based medicine live database that can be accessed at any time via mobile devices or the hospital network.

#### Recommendation 6.2 We suggest that medical institutions embed the evidence-based medicine database of children's off-label drugs into the hospital's electronic medical record system to enhance evidence-based decision-making, practice, and management. (3; B)

Currently, awareness on the principles of adequate off-label drug use is poor among clinicians [61]. For half of the children who were prescribed drugs off-label there exist no records of the off-label use [62]. Therefore, we suggest to integrate the management of off-label use of drugs for children into the clinician's operating system [1, 63] so that the doctor can make records when prescribing the off-label drugs, can review the current evidence related to the off-label use of the particular drug and receive valuable feedback on drug-related information. We suggest to integrate off-label drug management into the pharmacist's drug management system [63], providing information support and assuring a standardized management process.

### Theme 7: monitoring and assessment of the risk of pediatric off-label use of drugs

#### Recommendation 7.1 We suggest that tertiary care hospitals with pediatric departments and pediatric specialty hospitals establish a multi-level ADR surveillance network to monitor the use of drugs in children through passive surveillance, active surveillance, and epidemiological studies. (5; C)

The World Health Organization [64] categorizes pharmacovigilance methods as passive surveillance (spontaneous reports and case series), active surveillance (sentinel institution surveillance, prescription data surveillance, and disease or drug registry studies), stimulated reporting, and observational studies (cross-sectional surveys, case–control studies, and cohort studies). ADR monitoring is carried out through various forms, including an adverse drug reaction probability scale [65] and an active surveillance network in countries other than China [66]. ADR monitoring primarily depends on data from the spontaneous submission method [67], but there is a severe problem with underreporting off-label use of drugs in China. As a result, we suggest that medical institutions investigate the possibility of establishing multi-level ADR monitoring networks based on domestic and international ADR monitoring experiences to implement multiple monitoring methods for ADRs.

#### Recommendation 7.2 We suggest that medical regulatory authorities construct an adverse drug reaction monitoring data system for off-label drug use in children to facilitate reporting of ADRs by parents. (4; C)

Currently, adverse drug response events are being monitored and reported through the National Adverse Drug Reaction Database of the State Drug Administration in China [68]. However, the database lacks monitoring and reporting of ADRs during the use of drugs beyond their respective drug instructions, and it primarily relies on the active reporting of medical institutions, making it unable to effectively track ADRs that happen outside medical facilities. The establishment of aparent-initiated reporting and registration after hospital diagnosis technique of monitoring children's ADRs was shown to be supported by 97% of parents [69]. We suggest gradually building and improving the monitoring system for adverse drug reactions in children, as well as adding a platform for parents of children to report adverse events. The purpose of establishing this method is to provide a foundation for the clinical application and management of off-label use of drugs in children, and to standardize the management process of ADRs to pediatric off-label use of drugs [70].

#### Recommendation 7.3 We suggest that the clinicians assess and monitor the adverse drug reactions in children who are prescribed off-label drugs in cooperation with the parents and guardians. (4; C)

While clinicians, pharmacists, and nurses should individually assess the risk of children taking off-label drugs during the process of monitoring adverse drug reactions, we suggest seeking help from parents or guardians of children, especially of children who are incapable of autonomous behavior [71, 72].

### Theme 8: patient education on the pediatric off-label use of drugs

#### Recommendation 8.1 For children who are not sufficiently autonomous, it is recommended to educate their guardians about the drugs used; for children with sufficient autonomy, drug education is suggested for both children and their guardians. (4; C)

Pediatricians should consider whether or not their child patients have the ability to be independent during drug education. 83.5% of medical working groups believed that the family members or guardians of children should be informed when they use off-label drugs [12]. As a result, we recommend providing comprehensive drug education to children and their guardians on the rationale for drug use, potential side effects, monitoring methods, and other issures related to the use of the drug [4, 73, 74].

#### Recommendation 8.2 When conducting education on off-label use of drugs in children, it is suggested that health care providers provide the children and/or their guardians with the drug information list of the drug in question, and explain the treatment plan, expected effects, and possible ADRs. (4; C)

Comprehensive drug education is essential to the safety of off-label use of drugs for children. For example, for children and adolescents who use psychotropic drugs beyond the label, a list of information on overdose drug instructions is required [75]. The drug education checklist should include the following items: the reason for the off-label use, indications, route of administration, the dose of administration, frequency of administration, precautions and possible adverse effects, and monitoring methods and follow-up time. In addition, clinicians should promptly communicate with the child and the family and fully inform them of the reasons for the off-label use of drugs and the possible ADRs and monitoring methods [75–80].

#### Recommendation 8.3 We suggest that children and their guardians be educated on drug knowledge using popularized material (such as animations or audiobooks). (4; C)

Effective drug education enhances doctor-patient communication, promotes public knowledge of safe drug use, and reduces misunderstanding-related doctor-patient confrontations [1, 81]. Because the public's understanding of off-label drug use is limited, clinical pharmacists and other medical professionals should provide targeted health education, considering the cultural or educational background of parents and their understanding of the disease, through education brochures, WeChat, mass media, and WeChat group chats, to make the guardians aware of the issues related to the safe use of the drugs [82–85]. Simultaneously, in order to increase children's knowledge acceptance, cognitive methods for children, such as animation and audiobooks, can be used to implement off-label drug education.

## Limitations

Although the guideline is the first Chinese evidence-based guideline for the management of pediatric off-label use of drugs, there were certain difficulties in the application of evidence-based methodology in the area of health care. Because of the diversity and heterogeneity of the types of research on each topic, we were unable to produce systematic reviews of evidence.

## Conclusion

Off-label use of drugs in children is common globally, also in China. This guideline provides 21 recommendations grouped into 8 themes and a list of evidence on the common types of off-label use of drugs in children, in order to assist the management of pediatricians, pharmacists, medical managers, policymakers, and primary care physicians on off-label use of drugs in pediatrics, as well as provide recommendations for future healthcare policies in China.

## Supplementary Information

**Additional file 1: Supplementary 1.** Management Guideline for the Off-label Use of Drugs in China (2021). **Supplementary 2.** A list of common types pediatric off-label use of drugs, evidence levels, and recommendations.

## Data Availability

The datasets used and/or analyzed during the current study are available from the first or corresponding author on reasonable request.

## Abbreviations

ADR: Adverse Drug Reactions
AGREE II: Appraisal of Guidelines for Research and Evaluation II
BNF-c: British National Formulary for Children
BRAT: PhRMA Benefit-Risk Action Team
CheckUp: Reporting Items for Updated Clinical Guidelines: Checklist for the Reporting of Updated Guidelines Checklist
EBMgt: Evidence-based Mmanagement Evaluation Tool
EMA: European Medicines Agency
FDA: Food and Drug Administration
GRADE: Grading of Recommendations Assessment, Development, and Evaluation
MERS: Middle Eeast Respiratory Syndrome Coronavirus
OCEBM: Oxford Center for Evidence-Based Medicine
PD: Pharmacodynamics
PK: Pharmacokinetics
RCT: Randomized Controlled Trial
RIGHT: Reporting Items for Practice Guidelines in Healthcare statement
SARS: Severe Acute Rrespiratory Syndrome
WHO: World Health Organization
